# Plasma neuropeptide levels in patients with schizophrenia, bipolar disorder, or major depressive disorder and healthy controls: A multiplex immunoassay study

**DOI:** 10.1002/npr2.12304

**Published:** 2022-11-22

**Authors:** Shinsuke Hidese, Fuyuko Yoshida, Ikki Ishida, Junko Matsuo, Kotaro Hattori, Hiroshi Kunugi

**Affiliations:** ^1^ Department of Psychiatry Teikyo University School of Medicine Itabashi‐ku Japan; ^2^ Department of Mental Disorder Research, National Institute of Neuroscience National Center of Neurology and Psychiatry Kodaira Japan; ^3^ Department of Behavioral Medicine, National Institute of Mental Health National Center of Neurology and Psychiatry Kodaira Japan; ^4^ Department of Psychiatry, National Center Hospital National Center of Neurology and Psychiatry Kodaira Japan; ^5^ Medical Genome Center National Center of Neurology and Psychiatry Kodaira Japan

**Keywords:** cognitive function, major psychiatric disorders, neuropeptide, symptom

## Abstract

**Aim:**

We aimed to compare neuropeptide levels between patients with major psychiatric disorders and healthy controls and examine their association with symptoms and cognitive function.

**Methods:**

The participants were 149 patients with schizophrenia, 115 patients with bipolar disorder (BD), 186 unremitted patients with major depressive disorder (MDD), and 350 healthy controls. Psychiatric (schizophrenic, manic, and depressive) symptoms, sleep state, and cognitive (premorbid intelligence quotient, general cognitive, and memory) functions were evaluated. A multiplex immunoassay kit was used to measure cerebrospinal fluid (CSF) and plasma α‐melanocyte‐stimulating hormone (MSH), β‐endorphin, neurotensin, oxytocin, and substance P levels.

**Results:**

The verification assay revealed that CSF α‐MSH, β‐endorphin, neurotensin, oxytocin, and substance P levels were too low to be reliably measured, while plasma α‐MSH, β‐endorphin, neurotensin, oxytocin, and substance P levels could be successfully measured. Plasma α‐MSH, β‐endorphin, neurotensin, oxytocin, and substance P levels were not significantly different between patients with schizophrenia, BD, or MDD and healthy controls. Plasma α‐MSH, β‐endorphin, neurotensin, oxytocin, and substance P levels were not significantly correlated with psychiatric symptom scores in patients with schizophrenia, BD, or MDD and cognitive function scores in patients or healthy controls.

**Conclusion:**

Our data suggest that plasma neuropeptide levels do not elucidate the involvement of neuropeptides in the pathology of schizophrenia, BD, or MDD.

## Abstract 2

Plasma α‐melanocyte‐stimulating hormone, β‐endorphin, neurotensin, oxytocin, and substance P levels showed no significant difference in any of patients with schizophrenia, bipolar disorder, or major depressive disorder compared to healthy controls. Our data suggest that plasma neuropeptide levels do not expound the involvement of neuropeptides in the pathology of schizophrenia, bipolar disorder, or major depressive disorder.

## Graphical abstract

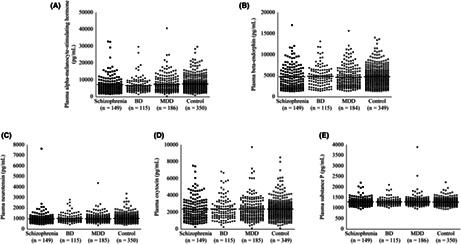

## 1 INTRODUCTION

Neuropeptides released in the brain and peripheral tissues serve as endocrine substances (e.g., hormones, inflammatory mediators, and neurotransmitters) through their G protein‐coupled receptors signaling,^1^, ^2^, ^3^ which has been suggested to be involved in the pathogenesis and pathophysiology of stress‐related neuropsychiatric disorders,^4^, ^5^ including schizophrenia^6^, ^7^, ^8^ and affective disorders.^9^, ^10^ Among neuropeptides, α‐melanocyte‐stimulating hormone (MSH), β‐endorphin, neurotensin, oxytocin, and substance P have been the focus of the present study.

Alpha‐MSH is related to anorexigenic function and systemic (i.e., peripheral and central) anti‐inflammatory and cyto‐protective processes,^11^, ^12^, ^13^, ^14^ which have been suggested to be involved in metabolic syndrome in patients with psychiatric disorders.^15^ Beta‐endorphin has been reported to have analgesic effects and reward‐centric and homeostasis‐restoring properties and has been suggested to be involved in the pathology of stress‐related psychiatric disorders,^16^ such as major depressive disorder (MDD).^17^ Neurotensin has been associated with immune and neuroendocrine modulation of enteric function,^18^ antinociception,^19^ and reward‐related behavior, especially in schizophrenia.^20^ Oxytocin has been related to antinociception, anxiety, feeding, social recognition, stress responses,^21^ and many other social behaviors,^22^, ^23^, ^24^ especially regarding social anxiety^25^ and socio‐emotional behavior in patients with psychiatric disorders.^26^ Substance P has been associated with pain perception^27^, ^28^ although its relationship with psychiatric disorders has not been well investigated.

Although the association between β‐endorphin and MDD pathophysiology has been suggested,^16^, ^17^ such association was tested only in patients with schizophrenia showing that plasma β‐endorphin levels were higher in 43, 77, and 49 patients with schizophrenia than in 34, 74, and 47 healthy controls, respectively.^29^, ^30^, ^31^ A systematic review and meta‐analysis reported that cerebrospinal fluid (CSF) and plasma oxytocin levels were not altered in patients with schizophrenia, BD, or MDD compared with those in healthy controls,^32^ whereas the association of oxytocin with social anxiety or socio‐emotional behavior has been suggested in patients with schizophrenia and MDD.^25^, ^26^ However, plasma oxytocin levels were lower in 34 patients with schizophrenia than in 31 healthy controls,^33^ which was associated with poorer metacognition, but not with social cognition or neurocognition.^34^ Plasma oxytocin levels were lower in 30 patients with schizophrenia than in 21 controls and were positively associated with social cognition, processing speed, and working memory assessed by the MATRICS Consensus Cognitive Battery in patients.^35^ Plasma oxytocin levels were higher in 135 drug‐naïve patients with BD II than in 97 drug‐naïve patients with MDD and 119 healthy controls,^36^ while they were lower in 25 patients with BD II than in 29 controls.^37^ Plasma oxytocin levels were lower in 61 patients with MDD than in 60 healthy controls^38^ and positively correlated with overall quality of life, psychological health, and social relationships in 60 patients with MDD and 60 healthy controls.^39^ Plasma substance P levels were not different between 43 patients with schizophrenia and 34 healthy controls^31^ and between 42 medication‐naive patients with MDD and 57 healthy controls,^40^ which has become primary investigations for the association of substance P with schizophrenia and MDD.

Previous studies have focused on single neuropeptides (i.e., β‐endorphin, oxytocin, and substance P) with a relatively small sample size; however, the association of β‐endorphin with BD and MDD or that between substance P and BD has not been tested therein. To the best of our knowledge, CSF or plasma α‐MSH and neurotensin levels have not been measured in psychiatric disorders, while α‐MSH and neurotensin have been associated with metabolic syndrome in patients with schizophrenia, BD, and MDD^15^ and reward‐related behavior in patients with schizophrenia,^20^ respectively, and may have a potential role in the pathophysiology of the patients. Furthermore, recently developed multiplex immunoassays have never been applied to measure CSF or plasma neuropeptide levels in patients with psychiatric disorders, although they have been applied to six critical neuropeptides in the plasma of schizophrenia‐like mouse models.^41^

Using a multiplex immunoassay with a relatively large sample, we aimed to compare CSF and plasma levels of five neuropeptides (i.e., α‐MSH, β‐endorphin, neurotensin, oxytocin, and substance P) between patients with major psychiatric disorders and healthy controls and examine the association of neuropeptide levels with symptoms and cognitive function in patients and controls. We hypothesized that CSF and plasma neuropeptide levels would be altered in patients with schizophrenia, BD, or MDD compared with healthy controls and possibly be correlated with symptom or cognitive function scores in the patient and control groups.

## 2 METHODS

### 2.1 Participants

This study involved 149 patients with schizophrenia (mean age: 36.8 ± 10.5 years, 85 male and 64 female), 115 patients with BD (mean age: 40.8 ± 11.3 years, 54 male and 61 female), 186 unremitted cutoff score ≥7 in 21‐item Hamilton Depression Rating Scale (HAMD‐21) patients with MDD (mean age: 39.6 ± 10.8 years, 88 male and female), and 350 healthy controls (mean age: 40.5 ± 12.2 years, 172 male and 178 female) who were matched for sex and ethnicity (Japanese). The BD group included 38 patients with BD I and 77 with BD II. The total sample was not based on any power analyses because we did not have data on the pre‐obtained effect size. All participants were recruited at the National Center of Neurology (NCNP) via advertisements at the NCNP Hospital on our website and in local free magazines. Participants were screened for psychiatric disorders by trained psychiatrists using the Japanese version of the Mini International Neuropsychiatric Interview (M.I.N.I.).^42^, ^43^ Consensus diagnoses were determined according to the criteria laid out in the Diagnostic and Statistical Manual of Mental Disorders, 4th edition,^44^ based on information from the M.I.N.I., additional unstructured interviews, and medical records, if available. Healthy controls had no history of contact with psychiatric services. Participants were excluded if they had a history of central nervous system disease, severe head injury, substance abuse, or mental retardation. Written informed consent was obtained from every participant after they were provided a description of the study protocol. The study protocol was approved by the Ethics Committee of the NCNP. This study was conducted in accordance with the Declaration of Helsinki.^45^

### 2.2 Clinical assessments

The Positive and Negative Syndrome Scale (PANSS) was used to evaluate symptoms in patients with schizophrenia,^46^ and the Young Mania Rating Scale was used to evaluate manic symptoms in patients with BD.^47^ GRID HAMD‐21 was used to evaluate depressive symptoms in patients with BD and MDD.^48^ Trained psychiatrists assessed these symptoms. The Japanese version of the Pittsburgh Sleep Quality Index (PSQI) was used to evaluate sleep state.^49^ Premorbid intelligence quotient was evaluated using the face‐to‐face version of the Japanese Adult Reading Test (JART).^50^ General cognitive function was evaluated using the Japanese version of the Brief Assessment of Cognition in Schizophrenia (BACS).^51^, ^52^ Memory function was evaluated using the Japanese version of the Wechsler Memory Scale‐Revised (WMS‐R)^53^, ^54^ These cognitive functions were assessed by certified psychologists. Daily doses of antipsychotics were converted to chlorpromazine‐equivalent doses, and doses of antidepressants were converted to imipramine‐equivalent doses, according to published guidelines.^55^

### 2.3 Lumbar and venipunctures

CSF was collected as described in our previous multiplex immunoassay studies.^56^, ^57^ The plasma was collected in an ethylenediaminetetraacetic acid‐2 K contained tube (PK5, SRL Co. Ltd., Tokyo, Japan) and centrifuged (3000 rpm for 10 min or 2600 × *g* for 13 min) at 4°C and the supernatant was dispended in 1.5 mL or 0.6 mL aliquots and stored at −80°C in a deep freezer. Protease inhibitor was not added to plasma samples. After a single melting on ice for the sample set preparation (vortex and 12 000 rpm for 10 min at 4°C, and the supernatant was dispensed on 96‐well plates), it was refrozen at −80°C in a deep freezer until multiplex immunoassays were performed.

### 2.4 Multiplex immunoassays

Neuropeptide levels were measured with the MAGPIX CCD imaging system (Bio‐Rad Laboratories, Inc.) using a magnetic on‐bead antibody conjugation kit having species reactivity for human α‐MSH, β‐endorphin, neurotensin, oxytocin, and substance P (HNPMAG‐35 K, MILLIPLEX® MAP Human Neuropeptide Magnetic Bead Panel, Millipore, Inc.) according to the manufacturer's instructions. As a result of the verification assay in duplicate, CSF α‐MSH, β‐endorphin, neurotensin, oxytocin, and substance P levels were found to be lower than the assay working range; therefore, we decided to measure only plasma α‐MSH, β‐endorphin, neurotensin, oxytocin, and substance P levels which was found to be within the assay working range and use plasma samples diluted to at a ratio of 1:4. The assay was performed using single plasma samples to secure a large number after confirmation that the intra‐ and inter‐run coefficients of variance for the five neuropeptides accounted for less than 5% for intra‐run and 10% for inter‐run of the mean variance in the verification assay (intra‐run: maximum 3.8%, 1 set, duplicate; inter‐run: maximum 7.9%, 16 sets, duplicate).

A VIAFLO 96/384 system (INTEGRA Biosciences, Corp.) was used to simultaneously apply the samples to 96‐well plates. To adjust the inter‐assay variations between the 96‐well plates, 16 randomly selected plasma samples diluted at a ratio of 1:4 were used as margin samples to fit the measurements of nine plates to those of one standard plate that included eight standard dilutions and blank samples. Based on the measures of the margin samples, linear regression equations were calculated for the five neuropeptides using two‐dimensional scatter diagrams between the standard and the other nine plates to perform inter‐plate adjustment.

Among the five assayed neuropeptides, the measurements of neuropeptides satisfied the following three criteria and were deemed reliable: (i) within the assay working range, (ii) coefficients accounting for less than 5% of mean intra‐run (seven standard and one blank sample [duplicate]) and 20% of inter‐run variance (16 plasma samples [decuplicate]), and iii) strong Pearson's correlation coefficients (*r* > 0.7) in the linear regression equations of the inter‐plate adjustment. Plasma neuropeptide levels are reported in pg/mL. There were 6 missing values corresponding to 3 β‐endorphin (2 for MDD and 1 for healthy controls), 1 neurotensin (for MDD), and 2 oxytocin (1 for MDD and 1 for healthy controls) levels.

### 2.5 Statistical analyses

Categorical (i.e., sex and current smoking) and continuous (i.e., age, body mass index [BMI], and education) variables were compared between patients diagnosed with psychiatric disorders and healthy controls using chi‐square tests and Student's or Welch's *t*‐tests, respectively. PSQI, cognitive function (JART, BACS, and WMS‐R) scores, and plasma neuropeptide levels were compared between patients diagnosed with psychiatric disorders and healthy controls using multivariate analysis of covariance (MANCOVA), with adjustments for age, sex, BMI, education level, and current smoking status. Effect sizes were shown using Cramer's V for chi‐square tests, Cohen's d for unpaired *t*‐tests, and partial η^2^ for MANCOVAs.

Correlations between plasma neuropeptide levels and continuous clinical variables were assessed using Pearson's correlation coefficients, while plasma neuropeptide levels were compared between sexes and current smoking status using unpaired (Student's or Welch's) *t*‐tests. Correlations between plasma neuropeptide levels and symptoms and cognitive function scores were assessed using Pearson's partial correlation coefficient, with adjustments for age, sex, BMI, education level, current smoking status, and psychotropic medication use (only for patients). In addition, plasma neuropeptide levels were compared using MANCOVA between mood stabilizer and psychotropic medication use, with adjustments for age, sex, BMI, education level, and current smoking status. Correlation matrices for plasma neuropeptide levels were also assessed using Pearson's partial correlation coefficient, with adjustments for age, sex, BMI, education level, current smoking status, and psychotropic medication use (only for patients).

Bonferroni corrections for multiple testing were applied for group comparisons in t‐tests, chi‐square tests, MANCOVAs, and correlational analyses on the five plasma neuropeptide levels measured (*p* < 0.05/5 = 0.01). All statistical tests were two‐tailed, and *p* < 0.05 was considered significant. Statistical analyses were performed using Statistical Package for the Social Sciences version 28.0 (IBM Japan, Ltd., Tokyo, Japan).

## 3 RESULTS

Demographic and clinical characteristics of participants are presented in Table 1. Age was significantly lower in patients with schizophrenia than in healthy controls (corrected *p* < 0.05). BMI was significantly higher in patients with schizophrenia and those with BD than in healthy controls (corrected *p* < 0.05). Education level of patients with schizophrenia was significantly lower than in healthy controls (corrected *p* < 0.05). Rate of current smoking status was significantly higher in patients with schizophrenia and those with BD than in healthy controls (corrected *p* < 0.05). PSQI scores were significantly higher, whereas JART, all BACS, and all WMS‐R scores were significantly lower in patients with schizophrenia than in healthy controls (corrected *p* < 0.05). PSQI scores were significantly higher, whereas BACS verbal memory, working memory, motor speed, attention, and all WMS‐R scores were significantly lower in patients with BD than in healthy controls (corrected *p* < 0.05). PSQI scores were significantly higher, whereas BACS verbal memory, working memory, motor speed, attention, WMS‐R verbal memory, general memory, attention/concentration, and delayed recall scores were significantly lower in patients with MDD than in healthy controls (corrected *p* < 0.05).

**TABLE 1:** Demographic and clinical characteristics of participants

	Schizophrenia (*n* = 149)			Statistical comparisons	Bipolar disorder (*n* = 115)			Statistical comparisons	Major depressive disorder (*n* = 186)			Statistical comparisons	Healthy control (*n* = 350)		
	Mean ± Standard deviation			vs control	Mean ± Standard deviation			vs control	Mean ± Standard deviation			vs control	Mean ± Standard deviation		
Age (years)	36.8	±	10.5	Welch's *t* = 3.44, ***p* = 6.5.E‐4**, Cohen's d = 0.32	40.8	±	11.3	Student's *t* = −0.24, *p* = 0.82, Cohen's d = −0.03	39.6	±	10.8	Welch's *t* = 0.88, *p* = 0.38, Cohen's d = 0.08	40.5	±	12.3
Sex, men (%)	85 (57.0)			χ^2^ = 2.61, *p* = 0.11, Cramer's V = 0.07	54 (47.0)			χ^2^ = 0.17, *p* = 0.68, Cramer's V = 0.02	88 (47.3)			χ^2^ = 0.16, *p* = 0.69, Cramer's V = 0.02	172 (49.1)		
Body mass index (kg/m^2^)	24.3	±	4.9	Welch's *t* = −4.39, ***p* = 1.8.E‐5**, Cohen's d = −0.49	23.8	±	4.6	Welch's *t* = −3.15, ***p* = 0.0020**, Cohen's d = −0.39	22.8	±	4.3	Welch's *t* = −1.21, *p* = 0.23, Cohen's d = −0.12	22.3	±	3.4
Education (years)	13.8	±	2.4	Welch's *t* = 5.31, ***p* = 2.4.E‐7**, Cohen's d = 0.54	15.1	±	2.6	Student's *t* = −0.55, *p* = 0.59, Cohen's d = −0.06	14.8	±	2.2	Student's *t* = 0.93, *p* = 0.35, Cohen's d = 0.09	15.0	±	2.1
Current smoking, n (%)	40 (26.8)			χ^2^ = 11.15, ***p* = 8.4.E‐4**, Cramer's V = 0.15	27 (23.5)			χ^2^ = 5.29, ***p* = 0.021**, Cramer's V = 0.11	33 (17.7)			χ^2^ = 1.11, *p* = 0.29, Cramer's V = 0.05	50 (14.3)		
Age of onset (years)	22.4	±	6.7		28.9	±	10.0		30.3	±	12.1				
Duration of illness (years)	14.2	±	9.4		11.2	±	7.8		6.8	±	5.7				
Chlorpromazine‐equivalent dose (mg/day)															
Total	498.3	±	752.8		89.5	±	226.4								
Typical	92.3	±	225.8		3.7	±	11.8								
Atypical	406.0	±	741.6		85.8	±	227.2								
Imipramine‐equivalent dose (mg/day)					60.3	±	115.1		86.1	±	122.1				
Mood stabilizer use, n (%)	27 (18.1)				80 (69.6)				28 (15.1)						
Psychotropic medication use, n (%)	131 (87.9)				97 (84.3)				121 (65.1)						
PANSS positive	13.7	±	4.9												
PANSS negative	16.0	±	6.3												
PANSS general psychopathology	30.2	±	8.4												
Young Mania Rating Scale					2.9	±	5.0								
21‐item Hamilton Depression Rating Scale					11.3	±	8.1		15.8	±	6.1				
Pittsburgh Sleep Quality Index	7.5	±	3.1	*F* = 60.01, ***p* = 5.5.E‐14**, partial η^2^ = 0.11	9.1	±	3.5	*F* = 161.59, ***p* = 6.4.E‐32**, partial η^2^ = 0.26	9.3	±	3.8	*F* = 237.65, ***p* = 1.5.E‐44**, partial η^2^ = 0.31	5.0	±	2.5
Japanese Adult Reading Test	73.7	±	15.7	*F* = 10.84, ***p* = 0.0011**, partial η^2^ = 0.02	76.8	±	13.4	*F* = 8.39, *p* = 0.0039, partial η^2^ = 0.02	80.1	±	11.7	*F* = 0.15, *p* = 0.70, partial η^2^ = 0.00	80.5	±	11.0
BACS verbal memory	40.8	±	13.2	*F* = 74.51, ***p* = 8.5.E‐17**, partial η^2^ = 0.13	44.3	±	11.3	F = 34.58, ***p* = 7.9.E‐9**, partial η^2^ = 0.07	47.1	±	10.5	*F* = 20.75, ***p* = 6.5.E‐6**, partial η^2^ = 0.04	50.5	±	9.6
BACS working memory	19.2	±	4.7	F = 41.99, ***p* = 2.2.E‐10**, partial η^2^ = 0.08	20.4	±	4.0	*F* = 14.01, ***p* = 2.0.E‐4**, partial η^2^ = 0.03	20.9	±	3.9	*F* = 11.80, ***p* = 6.4.E‐4**, partial η^2^ = 0.02	22.1	±	3.7
BACS motor speed	70.7	±	16.5	*F* = 54.84, ***p* = 5.7.E‐13**, partial η^2^ = 0.10	75.0	±	15.7	*F* = 32.73, ***p* = 1.9.E‐8**, partial η^2^ = 0.07	78.1	±	15.7	*F* = 13.30, ***p* = 2.9.E‐4**, partial η^2^ = 0.02	82.7	±	11.4
BACS verbal fluency	44.3	±	12.4	*F* = 14.18, ***p* = 1.9.E‐4**, partial η^2^ = 0.03	50.5	±	9.8	*F* = 5.25, *p* = 0.022, partial η^2^ = 0.01	48.3	±	10.8	*F* = 4.47, *p* = 0.035, partial η^2^ = 0.01	50.4	±	10.7
BACS attention	54.6	±	13.0	*F* = 135.92, ***p* = 6.8.E‐28**, partial η^2^ = 0.22	62.7	±	11.4	*F* = 35.38, ***p* = 5.4.E‐9**. partial η^2^ = 0.07	64.5	±	12.1	*F* = 26.84, ***p* = 3.2.E‐7**, partial η^2^ = 0.05	69.7	±	12.5
BACS executive function	16.8	±	3.5	*F* = 15.55, ***p* = 9.2.E‐5**, partial η^2^ = 0.03	17.7	±	2.7	*F* = 1.01, *p* = 0.31, partial η^2^ = 0.00	18.1	±	2.7	*F* = 0.10, *p* = 0.75, partial η^2^ = 0.00	18.0	±	2.6
WMS‐R verbal memory	92.1	±	19.1	*F* = 119.83, ***p* = 4.2.E‐25**, partial η^2^ = 0.20	103.0	±	17.5	*F* = 26.58, ***p* = 3.8.E‐7**, partial η^2^ = 0.06	108.1	±	14.7	*F* = 9.05, ***p* = 0.0029**, partial η^2^ = 0.02	112.1	±	15.0
WMS‐R visual memory	90.8	±	18.1	*F* = 63.76, ***p* = 1.0.E‐14**, partial η^2^ = 0.12	98.3	±	13.8	*F* = 27.40, ***p* = 2.5.E‐7**, partial η^2^ = 0.06	101.7	±	12.5	*F* = 7.18, *p* = 0.0066, partial η^2^ = 0.01	104.7	±	12.2
WMS‐R general memory	90.2	±	19.4	*F* = 134.97, ***p* = 9.8.E‐28**, partial η^2^ = 0.22	101.7	±	16.9	*F* = 34.44, ***p* = 8.5.E‐9**, partial η^2^ = 0.07	107.4	±	14.6	*F* = 8.96, ***p* = 0.0029**, partial η^2^ = 0.02	111.2	±	14.3
WMS‐R attention/concentration	97.5	±	15.3	*F* = 34.63, ***p* = 7.4.E‐9**, partial η^2^ = 0.07	100.5	±	13.0	*F* = 28.41, ***p* = 1.5.E‐7**, partial η^2^ = 0.06	103.6	±	15.0	*F* = 11.5, ***p* = 7.4.E‐4,** partial η^2^ = 0.02	108.3	±	13.6
WMS‐R delayed recall	88.4	±	19.0	*F* = 124.35, *p* = **6.8.E‐26**, partial η^2^ = 0.20	99.7	±	15.8	*F* = 31.68, ***p* = 3.2.E‐8**, partial η^2^ = 0.07	103.8	±	14.8	*F* = 14.68, *p* = **1.4.E‐4**, partial η^2^ = 0.03	108.7	±	14.2

*Note*: Corrected significant *p*‐values (*t*‐tests: *p* < 0.05/3 = 0.016, chi‐square tests: *p* < 0.05/2 = 0.025 multivariate analysis of covariance tests: *p* < 0.05/13 = 0.0038) are shown in bold cases.

Abbreviations: BACS, Brief Assessment of Cognition in Schizophrenia; PANSS, Positive and Negative Syndrome Scale; WMS‐R, Wechsler Memory Scale‐Revised.

**FIGURE 1:**
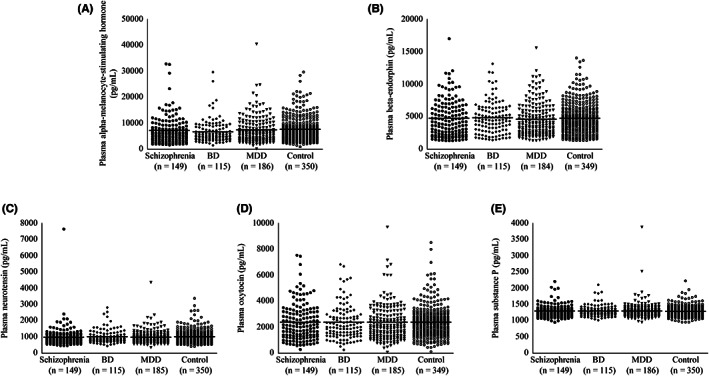
Dot plots of plasma neuropeptide levels in the four‐diagnostic groups. Plasma α‐melanocyte‐stimulating hormone (A), β‐endorphin (B), neurotensin (C), oxytocin (D), and substance P (E) levels in patients with schizophrenia, BD, or MDD and healthy controls. Five neuropeptide levels showed no significant difference in any of the three patient groups when compared to healthy controls. Horizontal lines in dotted plots denote mean values. Six missing values were corresponded to 3 β‐endorphin (MDD: *n* = 2 and healthy controls: *n* = 1), 1 neurotensin (MDD: *n* = 1), and 2 oxytocin (MDD: *n* = 1 and healthy controls: *n* = 1) levels. BD, bipolar disorder; MDD, major depressive disorder

Comparisons of plasma neuropeptide levels among the four diagnostic groups are presented in Table 2. No significant difference was observed in five neuropeptides (α‐MSH, β‐endorphin, neurotensin, oxytocin, and substance P) in patients with schizophrenia, BD, or MDD compared with healthy controls (Figure 1). Correlations between plasma neuropeptide levels and symptom scores are presented in Table 3. Plasma oxytocin and substance P levels were significantly and positively correlated with PSQI total scores in patients with schizophrenia (corrected *p* < 0.05), whereas there was no significant correlation with PANSS scores. No significant correlation was found among patients with BD, or MDD, or healthy controls. Correlations between plasma neuropeptide levels and cognitive function scores are presented in Table 4. No significant correlation was observed among patients with schizophrenia, BD, or MDD, or healthy controls.

**TABLE 2:** Comparisons of plasma neuropeptide levels among the four diagnostic groups

	Schizophrenia			Statistical comparisons	Bipolar disorder			Statistical comparisons	Major depressive disorder			Statistical comparisons	Healthy control		
	Mean ± Standard deviation			vs control	Mean ± Standard deviation			vs control	Mean ± Standard deviation			vs control	Mean ± Standard deviation		
α‐melanocyte‐stimulating hormone (pg/mL)	7375.0	±	5336.3	*p* = 0.40	6767.8	±	4361.3	*p* = 0.06	7456.5	±	5179.7	*p* = 0.56	7698.7	±	4707.6
β‐endorphin (pg/mL)	4768.6	±	2809.2	*p* = 0.80	4853.9	±	2467.2	*p* = 0.95	4672.3	±	2576.1	*p* = 0.45	4822.0	±	2419.2
Neurotensin (pg/mL)	1004.2	±	659.4	*p* = 0.96	1020.3	±	411.4	*p* = 0.99	993.1	±	436.8	*p* = 0.61	1016.2	±	399.3
Oxytocin (pg/mL)	2416.5	±	1423.5	*p* = 0.73	2381.6	±	1320.3	*p* = 0.95	2381.5	±	1378.4	*p* = 0.97	2381.3	±	1186.3
Substance P (pg/mL)	1299.0	±	202.1	*p* = 0.70	1304.1	±	174.8	*p* = 0.67	1308.8	±	262.4	*p* = 0.95	1293.6	±	156.3

**TABLE 3:** Correlations between plasma neuropeptide levels and symptom scores

	Schizophrenia								Bipolar disorder						Major depressive disorder				Healthy control	
	PANSS positive		PANSS negative		PANSS general		PSQI		Young mania rating scale		HAMD‐21		PSQI		HAMD‐21		PSQI		PSQI	
	*r*	*p*	*r*	*p*	*r*	*p*	*r*	*p*	*r*	*p*	*r*	*p*	*r*	*p*	*r*	*p*	*r*	*p*	*r*	*p*
α‐melanocyte‐stimulating hormone	−0.06	0.47	0.05	0.54	0.01	0.88	0.04	0.66	−0.14	0.14	0.01	0.95	0.05	0.57	−0.07	0.37	0.03	0.68	−0.01	0.89
β‐endorphin	0.01	0.89	0.10	0.25	0.08	0.36	0.15	0.06	−0.16	0.09	−0.11	0.27	0.02	0.82	−0.05	0.47	0.00	1.00	−0.01	0.78
Neurotensin	−0.06	0.45	0.14	0.10	0.07	0.39	0.03	0.68	−0.19	0.049	−0.01	0.88	0.01	0.91	−0.04	0.61	−0.01	0.91	−0.02	0.73
Oxytocin	−0.09	0.28	0.050	0.55	−0.01	0.87	0.24	**0.003**	−0.20	0.033	−0.07	0.44	−0.02	0.83	−0.06	0.39	−0.02	0.74	−0.01	0.88
Substance P	−0.08	0.35	0.06	0.49	0.01	0.88	0.22	**0.008**	−0.17	0.08	−0.03	0.73	0.05	0.57	−0.03	0.69	0.00	0.97	0.01	0.92

*Note*: Corrected significant *p*‐values (*p* < 0.05/5 = 0.01) are shown in bold cases.

Abbreviations: HAMD‐21, 21‐item Hamilton Depression Rating Scale; PANSS, Positive and Negative Syndrome Scale; PSQI, Pittsburgh Sleep Quality Index; *r*, Pearson's partial correlation coefficient.

**TABLE 4:** Correlations between plasma neuropeptide levels and cognitive function scores

	Japanese adult Reading test		BACS verbal memory		BACS working memory		BACS motor speed		BACS verbal fluency		BACS attention		BACS executive function		WMS‐R verbal memory		WMS‐R visual memory		WMS‐R general memory		WMS‐R attention/concentration		WMS‐R delayed recall	
	*r*	*p*	*r*	*p*	*r*	*p*	*r*	*p*	*r*	*p*	*r*	*p*	*r*	*p*	*r*	*p*	*r*	*p*	*r*	*p*	*r*	*p*	*r*	*p*
<Schizophrenia>																								
α‐MSH	−0.08	0.32	0.01	0.94	−0.02	0.84	0.00	0.99	−0.01	0.94	0.02	0.80	−0.11	0.21	0.02	0.78	−0.02	0.79	0.01	0.88	−0.08	0.34	0.02	0.82
β‐endorphin	−0.09	0.30	−0.02	0.78	0.01	0.89	−0.03	0.73	−0.05	0.54	−0.06	0.49	−0.12	0.14	−0.01	0.93	−0.04	0.66	−0.02	0.82	−0.07	0.43	0.02	0.84
Neurotensin	−0.05	0.58	−0.01	0.93	0.07	0.42	−0.14	0.09	−0.08	0.37	−0.05	0.52	−0.20	0.017	−0.01	0.95	−0.11	0.19	−0.04	0.62	−0.10	0.21	0.00	0.95
Oxytocin	−0.11	0.20	−0.01	0.87	−0.04	0.65	0.03	0.74	0.00	0.98	−0.03	0.68	−0.07	0.39	0.00	0.98	−0.07	0.39	−0.02	0.77	−0.03	0.75	0.05	0.53
Substance P	−0.10	0.21	−0.03	0.72	−0.02	0.80	−0.01	0.86	−0.04	0.67	−0.06	0.48	−0.11	0.18	0.01	0.90	−0.08	0.31	−0.02	0.82	−0.07	0.43	0.04	0.63
<Bipolar disorder>																								
α‐MSH	−0.01	0.92	−0.06	0.55	0.03	0.73	−0.16	0.09	0.06	0.55	0.09	0.37	0.07	0.46	0.00	0.98	0.07	0.47	0.01	0.89	−0.03	0.76	−0.01	0.92
β‐endorphin	0.10	0.30	0.01	0.96	0.08	0.38	−0.10	0.27	0.11	0.23	0.21	0.023	0.05	0.58	0.04	0.70	0.06	0.54	0.04	0.66	0.06	0.52	0.03	0.72
Neurotensin	0.01	0.95	0.05	0.63	0.03	0.76	−0.11	0.26	0.06	0.50	0.12	0.23	0.10	0.27	0.02	0.83	0.10	0.32	0.04	0.68	−0.04	0.69	0.02	0.82
Oxytocin	−0.02	0.83	0.01	0.95	0.00	0.98	−0.05	0.57	0.17	0.07	0.13	0.16	0.05	0.58	0.01	0.92	0.14	0.14	0.05	0.64	0.05	0.60	0.04	0.66
Substance P	0.05	0.62	0.02	0.87	0.06	0.52	−0.09	0.37	0.17	0.07	0.14	0.15	0.06	0.50	0.04	0.65	0.15	0.12	0.07	0.46	0.00	0.99	0.06	0.52
<Major depressive disorder>																								
α‐MSH	−0.06	0.41	−0.10	0.17	0.07	0.35	−0.08	0.29	−0.07	0.33	−0.04	0.64	0.01	0.92	−0.01	0.86	0.04	0.60	−0.04	0.61	0.06	0.44	0.03	0.64
β‐endorphin	0.01	0.89	−0.07	0.38	0.13	0.08	−0.07	0.35	−0.01	0.87	0.08	0.30	0.04	0.60	0.02	0.79	0.13	0.08	0.02	0.76	0.14	0.07	0.09	0.24
Neurotensin	0.01	0.91	0.00	0.97	0.08	0.27	−0.10	0.16	−0.01	0.90	0.03	0.68	0.07	0.35	0.06	0.40	0.12	0.10	0.06	0.40	0.09	0.23	0.11	0.13
Oxytocin	0.01	0.92	−0.01	0.87	0.11	0.13	−0.07	0.32	−0.03	0.66	0.01	0.89	0.04	0.64	0.05	0.47	0.13	0.07	0.05	0.51	0.10	0.20	0.12	0.09
Substance P	0.02	0.82	0.00	0.98	0.10	0.16	−0.07	0.34	−0.04	0.64	0.05	0.53	0.06	0.45	0.05	0.51	0.14	0.07	0.05	0.49	0.09	0.24	0.12	0.11
<Healthy control>																								
α‐MSH	0.01	0.82	−0.05	0.33	−0.01	0.85	0.04	0.45	0.04	0.51	−0.01	0.87	0.00	0.93	0.01	0.83	−0.03	0.53	0.00	0.98	0.00	0.96	−0.06	0.24
β‐endorphin	−0.02	0.67	−0.01	0.91	−0.04	0.46	0.02	0.76	−0.02	0.76	−0.03	0.54	0.02	0.72	0.02	0.67	−0.04	0.44	0.01	0.90	−0.01	0.89	−0.05	0.38
Neurotensin	−0.04	0.51	−0.02	0.76	0.01	0.88	0.05	0.38	−0.01	0.89	−0.03	0.60	0.04	0.49	0.02	0.71	−0.02	0.77	0.01	0.91	0.00	0.95	−0.03	0.63
Oxytocin	0.00	0.96	0.00	0.98	0.00	0.99	0.04	0.42	0.02	0.75	−0.06	0.25	0.01	0.91	0.02	0.66	−0.04	0.48	0.01	0.90	0.00	1.00	−0.04	0.50
Substance P	−0.02	0.65	−0.02	0.76	0.02	0.73	0.03	0.61	0.02	0.74	−0.03	0.57	0.02	0.67	0.02	0.71	−0.05	0.40	0.00	0.96	0.01	0.87	−0.05	0.38

Abbreviations: BACS, Brief Assessment of Cognition in Schizophrenia; MSH; melanocyte‐stimulating hormone; *r*, Pearson's partial correlation coefficient; WMS‐R, Wechsler Memory Scale‐Revised.

Correlations between plasma neuropeptide levels and continuous clinical variables are presented in Table S1. Plasma α‐MSH and neurotensin levels were significantly and positively correlated with education level in patients with BD (corrected *p* < 0.05). Comparisons of plasma neuropeptide levels among nominal clinical variables are presented in Table S2. Plasma α‐MSH levels were significantly higher in drug‐free patients with schizophrenia than in medicated patients with schizophrenia (corrected *p* < 0.05). Correlation matrices for plasma neuropeptide levels in patients with schizophrenia, BD, or MDD, and healthy controls are presented in Tables S3–S6, respectively. Significant and positive correlations were observed in all 10 pairs of two neuropeptides in all diagnostic groups (corrected *p* < 0.05).

## 4 DISCUSSION

The present study revealed that there was no significant difference in plasma α‐MSH, β‐endorphin, neurotensin, oxytocin, and substance P levels between 149 patients with schizophrenia, 115 patients with BD, and 186 patients with MDD and 350 healthy controls. Our findings on β‐endorphin and oxytocin are inconsistent with previous studies reporting higher plasma β‐endorphin levels in patients with schizophrenia (patient: *n* = 43, 77, and 49 vs control: *n* = 34, 74, 47),^29^, ^30^, ^31^ lower plasma oxytocin levels in patients with schizophrenia (patient: *n* = 34, 34, and 30 vs control: *n* = 31, 31, and 21),^33^, ^34^, ^35^ higher and lower plasma oxytocin levels in patients with BD II (patient: *n* = 135 and 25 vs control: *n* = 119 and 29),^36^, ^37^ and lower plasma oxytocin levels in patients with MDD (patient: *n* = 61 vs control: *n* = 60).^38^ In addition, a preclinical study reported increased neurotensin and oxytocin in genetic and/or environmental schizophrenia‐like mice using multiplex immunoassay.^41^ Our finding is based on more patients than those of most previous studies^29^, ^30^, ^31^, ^33^, ^34^, ^35^, ^37^, ^38^, ^40^ and rather support unaltered plasma oxytocin levels in schizophrenia, BD, or MDD in a systematic review and meta‐analysis^32^ or plasma substance P levels in patients with schizophrenia (patient: *n* = 43 vs control: *n* = 34)^31^ and patients with MDD (patient: *n* = 42 vs control: *n* = 57).^40^ Furthermore, to the best of our knowledge, this study is the first to show that plasma α‐MSH and neurotensin levels are not altered in patients with schizophrenia, BD, or MDD.

No significant correlation was found between psychiatric symptoms or cognitive function scores and plasma neuropeptide (i.e., α‐MSH, β‐endorphin, neurotensin, oxytocin, and substance P) levels in patients with schizophrenia, BD, or MDD, or healthy controls. These results may be reasonable considering that all five neuropeptides showed no significant difference between patient and control groups. The absence of association between psychiatric symptom scores and plasma oxytocin levels is inconsistent with previous studies reporting that plasma oxytocin levels negatively correlate with PANSS, especially prosocial, scores in 27 male and 23 female patients with schizophrenia^58^ and that plasma oxytocin levels negatively correlate with HAMD and State‐Anxiety Inventory scores in 25 patients with MDD.^59^ In addition, the absence of association between cognitive function scores and plasma oxytocin levels is consistent with a previous study reported that no association was observed between plasma oxytocin levels and neurocognition (*n* = 34),^34^ whereas it is somewhat inconsistent with the association of plasma oxytocin levels with processing speed and working memory (*n* = 30)^35^ in patients with schizophrenia. Considering that the number of patients in this study (schizophrenia: *n* = 149 and MDD: *n* = 186) was larger than those in previous reports,^34^, ^35^, ^58^, ^59^ the absence of an association of psychiatric symptoms or cognitive functions with plasma oxytocin levels may be more robust. However, positive correlations of the PSQI total score with plasma oxytocin and substance P levels were found in patients with schizophrenia. These results were unexplainable from well‐known functions of oxytocin^21^, ^22^, ^23^, ^24^, ^25^, ^26^ or substance P,^27^, ^28^ and the mechanisms remained elusive.

Unlike our hypotheses, our data showing the absence of alteration or correlation regarding five neuropeptides in patients with schizophrenia, BD, or MDD do not support previous reviews suggesting the involvement of α‐MSH,^15^ β‐endorphin,^16^, ^17^ neurotensin,^20^ and oxytocin^25^, ^26^ in the pathology of psychiatric disorders although metabolic syndrome, reward‐related behavior, and social anxiety or socio‐emotional behavior were not assessed in this study. Here, we could test such involvement using plasma samples, but not using CSF samples because CSF α‐MSH, β‐endorphin, neurotensin, oxytocin, and substance P levels were insignificant to be reliably measured. Unlike CSF neuropeptide levels, plasma neuropeptide levels do not reflect directly central nervous system neuropeptides concentrations; hence, the absence of association in this study may have been unavoidable. Considering previous preclinical^5^, ^9^, ^10^ and clinical^4^, ^6^, ^7^, ^8^ reviews, regional structural or functional brain alterations related to neuropeptides could occur in patients with psychiatric disorders although our data suggested that such alterations may not be reflected in plasma neuropeptide levels. Molecular imaging (e.g., positron emission and single photon emission computed tomography) studies targeting neuropeptides in the brain may explain the pathomechanism of neuropeptides in patients with psychiatric disorders, although such studies are still scarce in preclinical^60^, ^61^ and clinical stages.^62^, ^63^, ^64^

There are several limitations in this study. First, 87.9% of patients with schizophrenia, 84.3% those of BD, and 65.1% those of MDD were taking psychotropic medication, and the effects were adjusted in our correlational analyses. Second, our multiplex immunoassay results may have differed if plasma was collected in a tube containing protein stabilizer. Third, the interference of chronic somatic disorders such as diabetes, cardiovascular diseases, endocrinological problems, and their specific pharmacological treatment might have affected plasma neuropeptide levels, although the present cohort have not contained such information. Fourth, this study measured five neuropeptides using plasma samples, which resulted in the absence of statistical differences in the three patient groups (i.e., schizophrenia, BD, and MDD) compared to the control group; however, the other neuropeptides may become biomarkers for schizophrenia, BD, or MDD. Fifth, the study samples consist of three different psychiatric disorders (i.e., schizophrenia, BD, and MDD) but indeed the samples are not homogenic according to clinical state (e.g., age of onset, duration of illness, chlorpromazine or imipramine‐equivalent doses, and mood stabilizer use). Therefore, it may be difficult to draw univocal conclusions for all patients included to the study, generalizing the patients as major psychiatric disorders. Finally, this study was cross‐sectional; hence, we could not address the time‐course of changes in plasma neuropeptide level in each participant. Longitudinal studies may illuminate such changes and detect associations between neuropeptides and schizophrenia, BD, or MDD.

In conclusion, CSF α‐MSH, β‐endorphin, neurotensin, oxytocin, and substance P levels were be too low to be reliably measured. Plasma α‐MSH, β‐endorphin, neurotensin, oxytocin, and substance P levels were not different between patients with schizophrenia, BD, or MDD and healthy controls. Psychiatric symptoms or cognitive functions were not correlated with plasma α‐MSH, β‐endorphin, neurotensin, oxytocin, and substance P levels in patients with schizophrenia, BD, or MDD, or healthy controls. This study suggested that plasma neuropeptide levels cannot clarify the involvement of neuropeptides in the pathology of schizophrenia, BD, or MDD.

## AUTHOR CONTRIBUTIONS

S.H. and K.H. evaluated the symptoms and determined the diagnosis. I.I. and J.M. conducted the interviews and psychological assessments. S.H. selected and F.Y. prepared the plasma sample. K.H. and H.K. were involved in intellectual content. SH designed the study, performed laboratory experiments and statistical analyses, and wrote the manuscript, which was revised and approved by all authors.

## FUNDING INFORMATION

This work was supported by the Japan Society for the Promotion of Science KAKENHI (21 K07514, S.H.). This funding source was financially involved only in this study.

## CONFLICT OF INTEREST

The authors declare no conflict of interest.

## APPROVAL OF THE RESEARCH PROTOCOL BY AN INSTITUTIONAL REVIEWER BOARD

The study protocol was approved by the Ethics Committee of the NCNP.

## INFORMED CONSENT

Written informed consent was obtained from every participant.

## REGISTRY AND THE REGISTRATION

N/A.

## ETHICS STATEMENT

The Ethics Committee of the NCNP approved this study.

## Supporting information


Appendix S1

Table S1–S6

## Data Availability

The data that support the findings of this study are openly available in “raw_data” at doi: 10.1002/npr2.12304.
