# Supplementary material for: Plasma neuropeptide levels in patients with schizophrenia, bipolar disorder, or major depressive disorder and healthy controls: A multiplex immunoassay study
Source: Neuropsychopharmacol Rep. 2022 Nov 22;43(1):57–68. doi: 10.1002/npr2.12304 (PMC10009433; doi:10.1002/npr2.12304)
Supplement: Supplementary file 2 — Table S1–S6 [file NPR2-43-57-s001.pdf]

Table S1 Correlations between plasma neuropeptide levels and continuous clinical variables

	Age		Body mass index		Education		Age of onset		Duration of illness		CPeq total		CPeq typical		CPeq atypical		Imipramine-equivalent dose	
	r	p	r	p	r	p	r	p	r	p	r	p	r	p	r	p	r	p
<Schizophrenia>																		
α-MSH	-0.02	0.85	-0.07	0.41	0.03	0.70	0.15	0.08	-0.15	0.06	-0.11	0.18	0.04	0.65	-0.12	0.13		
β-endorphin	0.02	0.77	-0.04	0.61	-0.03	0.73	0.12	0.14	-0.08	0.32	-0.10	0.22	0.04	0.66	-0.11	0.16		
Neurotensin	0.08	0.33	0.08	0.32	-0.05	0.55	0.08	0.33	0.01	0.91	-0.10	0.25	-0.02	0.78	-0.09	0.28		
Oxytocin	0.06	0.47	-0.04	0.64	-0.04	0.59	0.14	0.10	-0.07	0.42	-0.11	0.19	0.09	0.28	-0.14	0.09		
Substance P	0.09	0.28	-0.02	0.84	-0.06	0.49	0.13	0.12	-0.02	0.78	-0.07	0.41	0.09	0.28	-0.10	0.24		
<Bipolar disorder>																		
α-MSH	0.00	0.99	0.00	0.97	0.26	**0.005**	0.00	0.98	0.02	0.82	0.05	0.56	-0.03	0.72	0.06	0.55	0.02	0.87
β-endorphin	0.02	0.80	-0.01	0.88	0.21	0.021	-0.01	0.93	0.07	0.44	0.16	0.09	-0.01	0.93	0.16	0.09	0.05	0.62
Neurotensin	0.07	0.48	0.01	0.93	0.27	**0.004**	0.04	0.69	0.07	0.48	0.04	0.68	-0.07	0.47	0.04	0.65	0.00	0.96
Oxytocin	0.08	0.37	0.03	0.77	0.18	0.06	0.03	0.75	0.10	0.29	0.03	0.76	0.02	0.83	0.03	0.77	-0.04	0.70
Substance P	0.05	0.62	-0.04	0.65	0.20	0.031	-0.02	0.82	0.09	0.34	0.05	0.58	0.02	0.82	0.05	0.59	-0.03	0.79
<Major depressive disorder>																		
α-MSH	-0.08	0.25	0.08	0.27	0.07	0.32	-0.05	0.51	0.14	0.06							-0.02	0.78
β-endorphin	-0.01	0.91	0.06	0.39	0.09	0.24	0.00	0.97	0.07	0.33							0.02	0.77
Neurotensin	0.02	0.75	0.07	0.37	0.06	0.42	0.01	0.95	0.13	0.08							0.03	0.70
Oxytocin	0.02	0.80	0.09	0.20	0.05	0.49	-0.02	0.77	0.11	0.12							0.02	0.84
Substance P	0.05	0.48	0.02	0.79	-0.02	0.78	0.06	0.39	0.04	0.59							-0.01	0.86
<Healthy control>																		
α-MSH	-0.11	0.038	-0.04	0.43	-0.02	0.76												
β-endorphin	-0.08	0.15	-0.04	0.44	-0.06	0.24												
Neurotensin	0.00	0.95	0.00	0.95	-0.09	0.08												
Oxytocin	-0.04	0.41	-0.01	0.82	-0.03	0.56												
Substance P	0.00	0.99	0.00	0.95	-0.07	0.20												

CPeq, chlorpromazine-equivalent dose; MSH; melanocyte stimulating hormone

r, Pearson's correlation cofficient

Correctedly significant p-values are shown in bold cases ($p < 0.01$).

Table S2 Comparisons of plasma neuropeptide levels among nominal clinical variables

	Sex		Current smoking		Mood stablizer use		Psychotropic medication use	
	t	p	t	p	F	p	F	p
<Schizophrenia>								
α-MSH	-0.88	0.38	1.22	0.23	1.01	0.31	-2.66	**0.009**
β-endorphin	-0.28	0.78	0.86	0.39	-0.29	0.77	-1.21	0.24
Neurotensin	-1.24	0.22	0.71	0.48	0.84	0.40	-1.09	0.29
Oxytocin	-0.78	0.43	1.51	0.13	0.91	0.37	-0.98	0.33
Substance P	-0.59	0.55	1.20	0.23	0.77	0.44	-0.88	0.38
<Bipolar disorder>								
α-MSH	-1.84	0.07	-1.54	0.13	1.23	0.22	0.55	0.58
β-endorphin	-1.33	0.19	-0.36	0.72	0.35	0.73	1.22	0.22
Neurotensin	-1.22	0.22	-0.34	0.74	1.27	0.21	0.34	0.73
Oxytocin	-1.12	0.26	-0.92	0.36	1.12	0.26	0.39	0.69
Substance P	-1.01	0.31	-1.43	0.16	1.04	0.30	0.99	0.32
<Major depressive disorder>								
α-MSH	-1.05	0.30	1.01	0.31	-1.02	0.32	-0.21	0.84
β-endorphin	-1.22	0.22	1.18	0.24	-0.76	0.46	0.00	1.00
Neurotensin	-1.11	0.27	0.58	0.56	-1.09	0.29	0.16	0.87
Oxytocin	-1.38	0.17	0.90	0.37	-0.44	0.66	-0.39	0.70
Substance P	-1.97	0.050	0.88	0.38	-0.39	0.69	0.25	0.80
<Healthy control>								
α-MSH	-1.82	0.07	1.11	0.27				
β-endorphin	-1.62	0.11	1.94	0.06				
Neurotensin	-2.49	0.013	2.00	0.048				
Oxytocin	-0.34	0.73	1.71	0.09				
Substance P	-0.82	0.41	1.03	0.30				

MSH; melanocyte stimulating hormone

A correctedly significant p-value is shown in a bold case ($p < 0.01$).

Table S3 Correlation matrix for plasma neuropeptide levels in patients with schizophrenia

	α-MSH		β-endorphin		Neurotensin		Oxytocin		Substance P	
	r	p	r	p	r	p	r	p	r	p
α-MSH	-	-	0.81	**1.8.E-34**	0.70	**2.0.E-22**	0.77	**1.7.E-29**	0.83	**2.2.E-38**
β-endorphin			-	-	0.75	**1.6.E-27**	0.87	**1.1.E-45**	0.90	**4.6.E-55**
Neurotensin					-	-	0.60	**6.3.E-16**	0.72	**9.2.E-25**
Oxytocin							-	-	0.95	**8.1.E-74**
Substance P									-	-

MSH; melanocyte stimulating hormone

r, Pearson's partial correlation cofficient

Correctedly significant p-values are shown in bold exponents ($p < 0.01$).

Table S4 Correlation matrix for plasma neuropeptide levels in patients with bipolar disorder

	α-MSH		β-endorphin		Neurotensin		Oxytocin		Substance P	
	r	p	r	p	r	p	r	p	r	p
α-MSH	**-**	**-**	0.80	**2.1.E-26**	0.84	**1.5.E-31**	0.76	**9.5.E-23**	0.83	**7.6.E-30**
β-endorphin			**-**	**-**	0.82	**2.3.E-28**	0.82	**7.7.E-28**	0.84	**1.6.E-30**
Neurotensin					**-**	**-**	0.83	**4.0.E-30**	0.85	**7.1.E-32**
Oxytocin							**-**	**-**	0.91	**1.1.E-44**
Substance P									**-**	**-**

MSH; melanocyte stimulating hormone

r, Pearson's partial correlation cofficient

Correctedly significant p-values are shown in bold exponents (p < 0.01).

Table S5 Correlation matrix for plasma neuropeptide levels in patients with major depressive disorder

	α-MSH		β-endorphin		Neurotensin		Oxytocin		Substance P	
	r	p	r	p	r	p	r	p	r	p
α-MSH	-	-	0.79	**1.6.E-39**	0.81	**3.3.E-43**	0.73	**2.6.E-31**	0.76	**1.2.E-34**
β-endorphin			-	-	0.84	**2.4.E-49**	0.88	**1.4.E-60**	0.87	**3.7.E-58**
Neurotensin					-	-	0.85	**1.5.E-50**	0.87	**7.2.E-56**
Oxytocin							-	-	0.94	**4.0.E-88**
Substance P									-	-

MSH; melanocyte stimulating hormone

r, Pearson's partial correlation cofficient

Correctedly significant p-values are shown in bold exponents ($p < 0.01$).

Table S6 Correlation matrix for plasma neuropeptide levels in healthy controls

	α-MSH		β-endorphin		Neurotensin		Oxytocin		Substance P	
	r	p	r	p	r	p	r	p	r	p
α-MSH	-	-	0.77	**9.8.E-68**	0.76	**1.7.E-66**	0.75	**8.4.E-65**	0.76	**1.0.E-67**
β-endorphin			-	-	0.85	**2.7.E-96**	0.85	**4.4.E-100**	0.88	**4.2.E-115**
Neurotensin					-	-	0.79	**1.6.E-76**	0.82	**4.0.E-87**
Oxytocin							-	-	0.95	**8.4.E-170**
Substance P									-	-

MSH; melanocyte stimulating hormone

r, Pearson's partial correlation cofficient

Correctedly significant p-values are shown in bold exponents ($p < 0.01$).
